# A rare presentation of hepatocellular carcinoma in non-cirrhotic liver

**DOI:** 10.11604/pamj.2017.28.69.13512

**Published:** 2017-09-22

**Authors:** Lamia Kabbage, Meryem El Kouhen, Ahmed Taghy, Kaoutar Znati, Nawal Kabbaj

**Affiliations:** 1 Faculty of medicine, mohammed V Souissi University Rabat Morocco; 2 EFD-hepatogastroenterology Unit, ibn sina hospital, Rabat,Morocco; 3 Clinique chirurgicale B, ibn sina hospital, Rabat, Morocco; 4 Pathology departement, ibn sina hospital, Rabat, Morocco

**Keywords:** Hepatocellular carcinoma, GIST, non cirrhotic liver

## Abstract

Hepatocellular carcinoma is the most frequent type of liver malignancy. Most cases of hepatocellular carcinoma are secondary to either viral hepatitis (hepatitis B, C) or alcoholic cirrhosis. Liver cirrhosis due to any other causes is considered as a risk factor for development of hepatocellular carcinoma; however, hepatocellular carcinoma in non cirrhotic livers remains a rare condition. The present case report describes a 59-year-old woman patient admitted to explore right hypochondriac and epigastric pain, with no evidence of pre-existing liver disease and with a good general condition. The computed tomography was very suggestive of a gastro-intestinal stromal tumor. But, at laparotomy, a huge hepatic tumor was discovered. Histopathological study confirmed the presence of primary hepatocellular carcinoma. Hepatocellular carcinoma occurs more frequently on a cirrhotic liver. However, it can occur on a non cirrhotic liver and remains and extremely rare case.

## Introduction

The incidence of hepatocellular carcinoma (HCC) is constantly rising throughout the world with the majority of cases in Asia and Africa due to the high prevalence of hepatitis B virus (HBV) infection [1]. HCC is the most frequent type of primary malignant lesion of the liver, the sixth leading cause of cancer and the third leading cause of cancer death [2]. It develops on cirrhotic liver in 80% of cases, appearing in a 20% on healthy livers (fibrolamellar variant) or with non-cirrhotic chronic liver disease [3]. Besides this fibrolamellar variant, HCC on a healthy liver remains exceptional [4, 5]. We report a case of an HCC on a healthy liver diagnosed initially as a gastro-intestinal stromal tumor (GIST).

## Patient and observation

**Figure 1:**
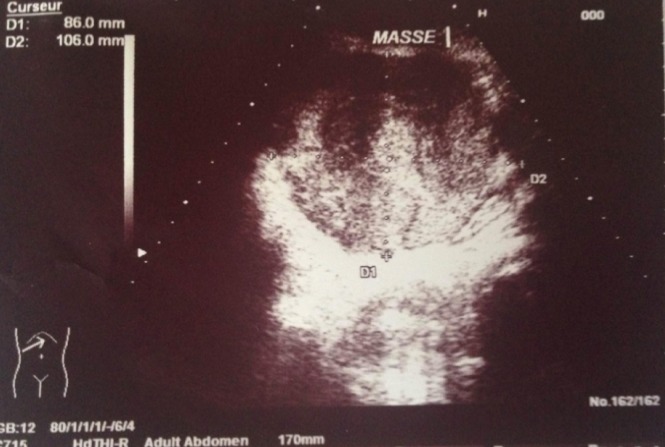
Abdominal ultrasonography: heterogeneous masse probably depending of the right hepatic lobe

**Figure 2:**
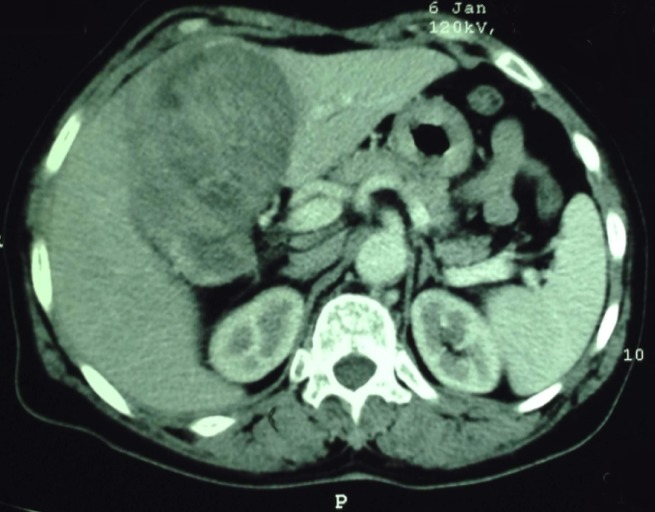
Abdominal CT: heterogeneous hepatic masse misdiagnosed as GIST

**Figure 3:**
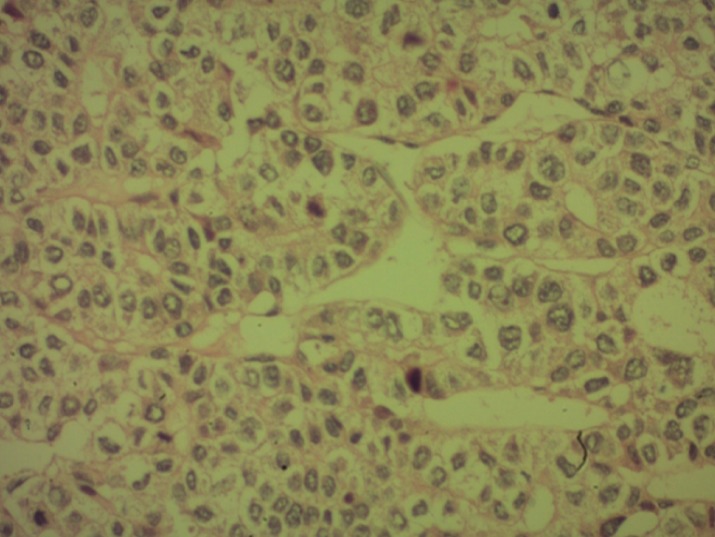
Histopathological study of liver biopsy with well differenciated cells consistent with HCC

A 59-year-old woman without pathological antecedents presented with a 2 months history of right hypochondriac and epigastric pain. She gave no history of jaundice or gastro-intestinal bleeding. She had a good general well being. On examination, the patient was not jaundiced and the body mass index (BMI) was in the standards (BMI = 24.6kg/m^2^). The abdominal examination revealed an enormous and firm mass of the right hypochondriac that reaches the right iliac fosse, no splenomegaly or ascites. She had no peripherical stigmata of chronic liver disease. Examination of other systems was unremarkable. Abdominal ultrasound was done and showed a hypodense and heterogeneous lesion of 10.6cm in diameter which could be located in the right hepatic lobe (doubt with a space tumor); otherwise, there were no signs of portal hypertension or ascites (Figure 1). An abdominal computed tomography (CT) revealed a tissular lesion of 151 x 108 x 82 mm with heterogeneous enhancement, located in the right flank having an intimate contact with the gastric antrum (Figure 2). It develops towards the hepatic hilus and compresses the biliay convergence inducing a slight intrahepatic bile ducts dilatation. No lymph nodes or ascites or signs of cirrhosis and portal hypertension were noticed. Therefore, the CT aspect was very suggestive of a GIST. The upper gastro-intestinal endoscopy revealed an extrinsic compression aspect in the antopyloric junction but did not show an endoluminal tumor. The liver tests showed minimum pattern of cholestasis: AP: 132UI/L (< 110), GGT: 32UI/L (< 50), total bilirubine: 31.2 mg/l (< 10) and a minimum increase in transaminases: GOT: 65UI/L (< 36), GPT: 90UI/L (< 34). Cell blood count (CBC) revealed a Hb of 10.8 gm/dL, WBC of 4800/cmm and a platelets of 160000/cm. The prothrombin level was 78%. Serology for viral hepatitis including HBsAg, HBcAb, HBsAb and HVC-Ab was negative. The patient underwent surgery for GIST resection. At laparotomy, the surgeon discovered an enormous hepatic tumor of 15cm in diameter, polylobed with dented contours, friable with necrotic foci and located in segments IV, V and VIII. The tumor was locally advanced, having a tight adherence with the gastric antrum, the first jejunal loop and omentum adherence. The tumor also invaded the gallbladder and the hepatic pedicle. Unfortunately, the tumor resection could not be done. Cirrhosis and liver chronic disease were rule out with a biopsy of the non-tumoral liver. The histopathological study of the surgical biopsy of the tumor revealed a well differentiated HCC (Figure 3). There were no eosinophilic, polygonal cells or wide lamellar bands of fibrous tissue that are characteristic of the fibrolamellar HCC. Since the tumor was unresectable, the patient was proposed for a treatment by sorafenib. Unfortunately, by lack of means, our patient could not have the chemotherapy by Sorafenib and she died after 5 months of HCC diagnosis.

## Discussion

HCC is the common type of primary liver cancer. It has an annual incidence of 600,000 newly diagnosed patients [6]. HCC in non cirrhotic liver is a relatively rare diagnosis: 14% in Schutte's cohort (664 cases of HCC) [7], 8.8% in Gomez-Rodriuez's cohort (469 cases of HCC) [8], 6.2% in Nunez-Martinez's cohort (469 cases of HCC) [9] and only 1.7% in Giannini's cohort including 3027 cases of HCC [10]. Some series of non cirrhotic HCC reported a lower male preponderance (male/female ratio: 1.3-2.1) as compared with the cirrhotic counterpart, where this ratio ranges from 3.2 to 8:1 [11]. The mean age is generally less advanced in non cirrhotic than in cirrhotic patients. Namely, cirrhotic HCC shows an unimodal age distribution, peaking at the 7^th^decade, whereas non cirrhotic HCC reveals a bimodal distribution, peaking at the 2^nd^ and 7^th^ decades [11]. In the presented case, the patient is a woman age of about sixty. Although liver cirrhosis is the main risk factor for HCC, this tumor may develop in patients with non cirrhotic chronic liver disease like chronic hepatitis B virus infection [12] and chronic hepatitis C virus infection [13, 14]. HCC can also develop in subjects without evidence of chronic liver disease. This condition remains exceptional and it represents only 0.32% in the literature's largest cohort of HCC including 3027 cases. This is compatible with the presented case as the patient showed no evidence of pre-existing liver disease. The pathogenesis is still not well understood. Several pathogenic risk factors are discussed such as ionizing radiation, exposure to toxins [5] and benign focal liver lesions, mainly the hepatocellular adenoma. Our patient had none of these risk factors (especially the hepatocellular adenoma). It can present as abdominal pain or discomfort in the right upper quadrant, jaundice, nausea, or “toxic syndrome” (weight loss, fever, malaise, asthenia, anorexia). Haemoperitoneum due to cancer rupture may be a life-threatening presentation of non cirrhotic HCC [11]. It may remain asymptomatic in more than half of cases explaining that it is often diagnosed at an advanced and inoperable stage. This was the case in this presentation. There was only a right hypochondriac and epigastric pain without any other associated symptoms. The patient had a good general condition. Therefore, the tumor was diagnosed at an advanced stage. Alpha-fetoprotein can be high or remain in the standards [15]. It exceeds 20 ng/dL less frequently in non cirrhotic than cirrhotic HCC patients (31-67% vs 59-84%) [11]. According to the guidelines, diagnosis is based on medical imagery and histology.

HCC in non cirrhotic patients shows similar CT and magnetic resonance imaging (MRI) patterns to those of HCC in cirrhotic patients. At CT, it demonstrates characteristic enhancement during the arterial phase and wash-out during the venous and/or equilibrium phases [16]. At MRI, it is hyperintense on T2-weighted images. On T1-weighted images, the lesion is hypointense [16]. HCC from non cirrhotic livers tended to be unifocal, large, well-circumscribed, encapsulated lesions or dominant masses with smaller satellite lesions [16]. Unfortunately, the abdominal CT performed in the presented case was very suggestive of a GIST and misdiagnosed the hepatic tumor. According to histological classification criteria of the World Health Organization (WHO), the trabecular type is the most frequent form (41-76%) in non-cirrhotic HCCs, as it is in cirrhotic subjects [11]. In our case, the histological study confirmed the diagnosis. The HCC was well differentiated and there were no characteristics of a fibrolamellar HCC. In the non tumoral liver, there were no signs of cirrhosis or chronic hepatitis (healthy liver). Patients with HCC diagnosed at an early stage are optimal candidates for resection, liver transplantation or percutaneous ablation. Surgical resection is recommended for patients with single tumors, absence of clinically relevant portal hypertension and normal bilirubin. Transplantation is indicated in patients with 3 nodules of < 3 cm or with single tumors < 5 cm with liver function impairment precluding resection. Transarterial chemoembolization (TACE) is recommended in asymptomatic patients with multinodular tumors that have not invaded hepatic vessels nor been disseminated outside the liver. Sorafenib is indicated as the first line of treatment in patients who cannot benefit from the above therapeutic options and still have a preserved liver function [17, 18]. In the case of our patient, the tumor was diagnosed at a very late stage. The size of the tumor and the severity of loco-regional invasion made the surgical resection impossible. According to the tumor stage and to the liver's biological parameters, the patient was a candidate for chemotherapy by sorafenib. Survival of patients with HCC in non cirrhotic liver mainly depends on tumor related factors such as tumor size, existence of satellite lesions, existence of a tumor capsule, vascular invasion, grading, R0 resection and the amount of intraoperative blood transfusions [7]. The altered condition, tobacco consumption, macroscopic vascular invasion, the large size of the tumor and the non surgical treatment were predictive factors of a bad prognosis of HCC on a healthy liver [19]. In the presented case, the tumor related factors and the absence of treatment worsened the prognosis. In fact, the patient died 5 months after the diagnosis of HCC.

## Conclusion

HCC on a healthy liver remains an exceptional condition. The pathogenesis is still not well understood. It is characterized by a clinical latency explaining the delay in the diagnosis and consequently, the poor prognosis at an advanced stage. Ultrasound monitoring; especially in patients with risk factors, finds its place in the early diagnosis of this exceptional tumor.

## Competing interests

The authors declare no competing interest.

## References

[1] El-Serag HB (2011). Hepatocellular carcinoma. N Engl J Med..

[2] Forner A, Ayuso C, Isabel Real M, Sastre J, Robles R, Sangro B (2009). Diagnosis and treatment of hepatocellular carcinoma. Med Clin (Barc)..

[3] Renedo F, De la Revilla J, Calleja JL (2008). Carcinoma hepatocelular. Medicine..

[4] Casanova-Martínez L, Castillo-Grau P, Jaquotot-Herranz M (2012). Hepatocellular carcinoma in non-cirrhotic liver. Rev Esp Enferm Dig..

[5] Singh P, Kaur H, Lerner RG, Patel R, Rafiyath SM, Singh Lamba G (2012). Hepatocellular carcinoma in non-cirrhotic liver without evidence of iron overload in a patient with primary hemochromatosis: review. J Gastrointest Cancer..

[6] Elmakki EE (2014). A Rare Presentation of Hepatocellular Carcinoma in a Young Adult: a case report. Oman Med J..

[7] Schütte K, Schulz C, Poranzke J, Antweiler K, Bornschein J, Bretschneider T (2014). Characterization and prognosis of patients with hepatocellular carcinoma (HCC) in the non-cirrhotic liver. BMC Gastroenterol..

[8] Gómez Rodríguez R, Romero Gutiérrez M, González de Frutos C (2011). Clinical characteristics, staging and treatment of patients with hepatocellular carcinoma in clinical practice: prospective study of 136 patients. Gastroenterol Hepatol..

[9] Núñez Martínez Ó, Matilla Peña A, Merino Rodríguez B (2011). Descriptive study of hepatocellular carcinoma in noncirrhotic liver. Gastroenterol Hepatol..

[10] Giannini EG, Marenco S, Bruzzone L, Savarino V, Farinati F (2012). Hepatocellular carcinoma in patients without cirrhosis in Italy. Dig Liver Dis..

[11] Trevisani F, Frigerio M, Santi V, Grignaschi A, Bernardi M (2010). Hepatocellular carcinoma in non-cirrhotic liver: a reappraisal. Dig Liver Dis..

[12] Wang Q, Luan W, Villanueva GA, Rahbari NN, Yee HT, Manizate F, Hiotis SP (2012). Clinical prognostic variables in young patients (under 40 years) with hepatitis B virus-associated hepatocellular carcinoma. J Dig Dis..

[13] Nosotti L, D'Arca T, Marignani M, Balducci G (2011). Hepatocellular Carcinoma in a non-cirrhotic liver of a HCV-Positive Woman with sustained viral response. Mediterr J Hematol Infect Dis..

[14] Nash KL, Woodall T, Brown AS, Davies SE, Alexander GJ (2010). Hepatocellular carcinoma in patients with chronic hepatitis C virus infection without cirrhosis. World J Gastroenterol..

[15] Alkofer B, Lepennec V, Chiche L (2011). Hepatocellular cancer in the non-cirrhotic liver. J Visc Surg..

[16] Di Martino M, Saba L, Bosco S, Rossi M, Miles KA, Di Miscio R (2014). Hepatocellulat carcinoma (HCC) in non-cirrhotic liver: clinical, radiological and pathological findings. Eur Radiol..

[17] Meloni TF, Di Stasi M, Rolle E, Solbiati L, Tinelli C (2008). Sustained complete response and complications rates after radiofrequency ablation of very early hepatocellular carcinoma in cirrhosis: Is resection still the treatment of choice. Hepatology..

[18] Llovet JM, Ricci S, Mazzaferro V, Hilgard P, Gane E, Blanc JF (2008). SHARP investigators study group: sorafenib in advanced hepatocellular carcinoma. N Engl J Med..

[19] Wörns MA, Bosslet T, Victor A, Koch S, Hoppe-Lotichius M, Heise M, Hansen T, Pitton MB, Niederle IM, Schuchmann M, Weinmann A, Düber C, Galle PR, Otto G (2012). Prognostic factors and outcomes of patients with hepatocellular carcinoma in non-cirrhotic liver. Scand J Gastroenterol..

